# On validation and invalidation of biological models

**DOI:** 10.1186/1471-2105-10-132

**Published:** 2009-05-07

**Authors:** James Anderson, Antonis Papachristodoulou

**Affiliations:** 1 Doctoral Training Centre, University of Oxford, Wolfson Building, Parks Road, Oxford, OX1 3QD, UK; 2 Department of Engineering Science, University of Oxford, Parks Road, Oxford, OX1 3PJ, UK; 3 Oxford Centre for Integrative Systems Biology, South Parks Road, Oxford, OX1 3QU, UK

## Abstract

**Background:**

Very frequently the same biological system is described by several, sometimes competing mathematical models. This usually creates confusion around their validity, ie, which one is correct. However, this is unnecessary since validity of a model cannot be established; model validation is actually a misnomer. In principle the only statement that one can make about a system model is that it is incorrect, ie, invalid, a fact which can be established given appropriate experimental data. Nonlinear models of high dimension and with many parameters are impossible to invalidate through simulation and as such the invalidation process is often overlooked or ignored.

**Results:**

We develop different approaches for showing how competing ordinary differential equation (ODE) based models of the same biological phenomenon containing nonlinearities and parametric uncertainty can be invalidated using experimental data. We first emphasize the strong interplay between system identification and model invalidation and we describe a method for obtaining a lower bound on the error between candidate model predictions and data. We then turn to model invalidation and formulate a methodology for discrete-time and continuous-time model invalidation. The methodology is algorithmic and uses Semidefinite Programming as the computational tool. It is emphasized that trying to invalidate complex nonlinear models through exhaustive simulation is not only computationally intractable but also inconclusive.

**Conclusion:**

Biological models derived from experimental data can never be validated. In fact, in order to understand biological function one should try to invalidate models that are incompatible with available data. This work describes a framework for invalidating both continuous and discrete-time ODE models based on convex optimization techniques. The methodology does not require any simulation of the candidate models; the algorithms presented in this paper have a worst case polynomial time complexity and can provide an exact answer to the invalidation problem.

## Background

Mathematical modelling is the new key tool in systems biology [1]: There now exist multiple differential equation models for a wide range of biological phenomena, sometimes over multiple time and spatial scales, from the molecular to the systems level. Depending on the system under study, models [2] can be in the form of discrete-time or continuous-time Ordinary Differential Equations (eg, chemical reaction networks with mass action kinetics), Functional (Delay) Differential Equations (eg, to describe maturation/growth in population dynamics), Stochastic Differential Equations (eg, to model chemical reaction networks in which species are found in low copy numbers), Partial Differential Equations (eg, to describe spatial dynamics), or even Hybrid models (which incorporate both discrete and continuous states) to model genetic networks. Central to all of these models are the inherent non-linearities that real life systems exhibit, which makes their analysis more complicated.

Models are usually developed based on physical principles which constrain their structure; typically these models contain parameters that are identified (fitted) using appropriate sets of observed experimental data and computational techniques [3-5]. No two experiments will ever yield exactly the same data even when carried out under a strict protocol, but one hopes that the model can explain the data within experimental error – hence it is claimed that the model is *valid*. In fact, to validate a model would require an infinite number of experiments and data [6,7]. Models that have many free parameters are usually prone for invalidation, as it is possible to fit a wide range of system responses if there are enough free parameters in the model. The claim that a model is valid is refuted when someone undertakes a different experiment which yields a new data set that cannot be explained by the model. Given this new data set, the model can be invalidated, by exhibiting the discrepancies between observed experimental data and the model behaviour. Model invalidation forms an integral part of the model development cycle, in which experiments are specifically designed in order to provide data sets that can be used to invalidate a subset of the models [5,8-10]. Also, model invalidation may help in identifying where parameters and/or system structure should be refined to reveal where the model is just incorrect.

Model validation has been studied from a control engineering perspective in [6,11], where the problem was posed in the robust control context: Given experimental input/output data and a model *P*, does there exist an external input (eg, noise) and set of system perturbations (*w*, Δ) such that the observed data are produced exactly? Here, the uncertainty in the model Δ is bounded and of a particular structure. Moreover, in [12] a methodology for validating continuous time models using finite experimental data is proposed.

Several other approaches have been developed using a statistical framework based on graphical residual analysis. Very frequently the *R*^2 ^statistic is used, which is a measure of the difference in response between model behaviour and data. Cross-validation can be used to test the capability of a model, constructed using a training set, to represent an unseen subset of the data, usually called the 'validation set' [3]. In this paper, we approach model invalidation from a different perspective. We first seek to answer the question 'How bad is the best model for this data set?', ie, to evaluate how good the model *structure *is to represent the experimental observations. To answer this question, recall that in parameter identification one seeks to find the point in the allowable parameter set that minimizes an objective function which encodes the requirement that the error between model evolution and data is small. (Sometimes this function is not convex and hence minimizing it using gradient descent algorithms may result in local minima [13].) While the objective is to identify a parameter choice in which the error is small, one should also pay attention to the dual question of what is the minimum of the possible error that could be obtained with the particular model structure. If this minimum error is unacceptably high, such a model may not be adequate to represent the experimental observations and a different one should be sought. In this paper, we develop an approach that can calculate a lower bound on this minimum error, which is important information to the user as for the validity of the model. For this purpose we use ideas based on the sum of squares decomposition of multivariate polynomials [14].

We then provide a methodology for discrete-time and continuous-time model invalidation using ideas for Real Algebraic Geometry and Semidefinite Programming. The aim is to construct functions/certificates that provide proof of the fact that the model can never represent an experimental data set. We stress that simulation cannot be used for this purpose, unless the data is certain, the model size is small and its structure, initial conditions and parameters are fixed. The reason for this is that as models become more complex (containing more states and parameters) exhaustive simulation for model invalidation becomes computationally prohibitive – as well as being inconclusive. Our methodology uses Semidefinite

Programming techniques which have been shown to have in the worst case a polynomial time complexity. For this purpose ideas presented in [15] are extended and a different class of functions is introduced. Related work appears in [16,17].

Ideas and notions related to model invalidation are reachability and systems verification; several different approaches have been developed in this area. For example, if one considers the experimental data set as the target set in a reachability analysis, then its *backwards reachable set *(ie, the set of states from which trajectories can reach the target set) can be used for model invalidation: if the backwards reachable set does not intersect the initial data set then the model can be invalidated. Mitchel et al., [18] propose a computationally tractable method for calculating the backward reachable set by solving the Hamilton-Jacobi-Isaacs partial differential equation.

The structure of this paper is as follows. In the next section we present some background material on the sum of squares decomposition of multivariate polynomials and how it can be obtained. Following this, we describe a method for obtaining a bound on the minimum achievable error between data and model structure as well as the methodology for invalidating continuous-time and then discrete-time models. Finally, we illustrate our results through a series of examples.

## Methods

In this section we formulate the way system models will be represented and introduce the sum of squares techniques that will be used in the sequel. We first introduce the following notation:

ℝ^*n *^*n*-dimensional real vector field

*N *number of sampled data points

 Sets describing the state space, initial conditions, state values at *t*_ℓ _and parameter values

*x*(*t*_*k*_) or *x*_*k *_predicted value of value of *x *∈ ℝ^*n *^at time *t*_*k *_from model

 or  experimental data at time *t*_*k*_

 (*t*_*k*_) or *i*^*th *^element of the data  taken at time *t*_*k*_

The final three terms have alternate notations as shown above and are used interchangeably where appropriate.

The models we consider are autonomous, in the form of Ordinary Differential Equations (ODEs) whose *n*-dimensional vector fields satisfy appropriate smoothness conditions in order to ensure that given an initial condition there exists a locally unique solution.

For *x *∈ ℝ^*n*^, let

(1)

be a candidate system model, where *p *∈  ⊂ ℝ^*m *^is a vector of parameters, such as kinetic constants etc. An alternative representation which is sometimes used is a discrete-time ODE of the form:

(2)

For the rest of the paper, we assume that either a model of the form (1) or of the form (2) is being considered, in which *f *is a polynomial function of its arguments. Experimental data (*t*_*i*_, ) for *i *= 1,..., *N *are provided, where  are the data points. The sets  encode the uncertainty in the data because of experimental error, etc. We will assume in the sequel that these sets are *semi-algebraic*, ie, they can be described by a finite set of polynomial inequalities. For example, if  for *i *= 1,..., *n *where  refers to the *i*^*th *^element of the experimental data taken at time *t*_1 _then we obtain the *n*-dimensional hypercube:

(3)

Note that when it is clear that we are talking about the whole vector we drop the superscript notation. In this paper we make repeated use of the notion of *sum of squares (SOS) polynomials*. A polynomial *p*(*y*), with real coefficients, where *y *∈ ℝ^*n*^, admits an SOS decomposition if one can find other polynomials *q*_1_,..., *q*_*m *_such that

(4)

where the subscripts denote the index of the *m *polynomials. If *p*(*y*) is SOS, it can be easily seen that *p*(*y*) ≥ 0 for all *y*, which means that *p*(*y*) is non-negative. Polynomial non-negativity is a very important property (as many problems in optimization and systems theory can be reduced to it) which is however very difficult to test (it has been shown to be NP-hard for polynomials of degree greater than or equal to 4 [19]). The existence of a SOS decomposition is a powerful relaxation for non-negativity – in fact, it can be verified in polynomial time. The reason is that *p*(*y*) being SOS is equivalent to the existence of a positive semidefinite matrix *Q *(ie, *Q *is symmetric and with non-negative eigenvalues) and a chosen vector of monomials *Z*(*y*) such that

(5)

A proof of this can be found in [14]. This essentially means that the SOS decomposition of *p*(*y*) can be computed using Semidefinite Programming. Software capable of formulating and solving the problem has been developed – SOSTOOLS [20]. All examples in this paper have been solved with SOSTOOLS and SeDuMi [21], a Semidefinite Programming solver. Semidefinite Programming has been used in the past for classification of complete parameter regions, see [22] for examples relevant to systems biology.

We now turn to the problems we wish to address. First, if a data set is provided, we develop a method for establishing how well the model structure, irrespective of the particular parameter values that one could choose, can represent the data. This can provide an indication for the quality of the model under consideration. Consequently, we consider the problem of model invalidation for continuous and discrete-time systems.

### How bad is the best model?

We begin this investigation by highlighting the link between system identification and model invalidation. The question we set out to answer is *"Given experimental data, what is the least error one can expect between the data and predictions from a model with the best parameter choice within the allowable parameter range?" *Most system identification questions try to find the best parameters in order to minimize an objective function of the error between model predictions and data, while the question we are asking here is dual to that: 'How bad is the best model, for all allowable parameters?' If the error is large, then this could indicate that the model structure may be inappropriate and one may want to invalidate the model and repeat the system identification cycle.

For the continuous time system (1), the scope of system identification is to find

(6)

where p* denotes the 'best' parameters and ||·|| denotes a norm on ℝ^*n *^(usually the 2-norm is used) and *N *the number of data points. The term  is many times obtained by simulation, but an appropriate approximation can also be used (eg, using the trapezoidal rule). Note that if the model is already in discrete-time, ie, its dynamics are described by (2), and if data are taken at the same discrete update points then the integral term does not need to be approximated. Either way, if this approximation results in an expression affine in the parameters *p*, and  then p* can be obtained via convex optimization (as the problem becomes convex). In this case, a simple gradient descent algorithm can be used to find the (global) minimum.

However, when the parameters do not enter in such a manner or when the experimental measurements are uncertain, then finding p* becomes more complicated and locally (but not globally) optimal parameter estimates may be obtained using optimization methods.

Instead, in this paper we develop a methodology for obtaining a lower bound on this error, ie, for finding *γ *such that



which can be used as an indication to whether the model structure that we are trying to fit is suitable or not. The reader is referred to the Discussion section for a description of the meaning of the value of *γ*. For this purpose we can use a method to approximate the integral term by setting *f*(*x*(*t*), *p*) = *f*(*x*_*i*_, *p*), which amounts to Euler discretization of the ODE (1), and then the left hand side of the equation above becomes



where *F *and *G *are polynomial functions of the parameter. Note that other discretization methods can also be used. Obtaining *γ *can be done efficiently by solving the following optimization problem:



The optimal value, p*, where p* ∈ ℝ^*m*^, can be obtained by the following simple procedure: Substitute *γ *into the expression *F*(*p*) - *γG*(*p*) and compute its SOS decomposition using SOSTOOLS. This gives a vector of monomials *Z*(*x*) and a positive semidefinite matrix *Q *as per (5). Applying some linear algebra we obtain a set of *m *simultaneous polynomial equations with *m *unknown variables in the form of (4), setting the right hand side of the system of equations to zero, i.e. *q*_1 _= *q*_2 _= ... = *q*_*m *_= 0 and solving produces the optimal parameter set p*.

We note that as the number of data points increases, so will the size of the error bound *γ *and a normalising factor should be introduced to facilitate comparison. The simplest method for achieving such a normalisation is to divide *γ *by the number of points, *N*. This will avoid favouring models fitted on smaller data sets to those fitted on larger ones.

When obtaining a bound on the error from the model it is unavoidable that the dataset will contain some uncertainty due to experimental error. Ignoring error in the data can be thought of as searching for a nominal model, while an appropriately defined parametric uncertainty accounts for the experimental error, a technique that underlies modern robust control theory [23,24]. If an estimate of the error in the data is known a priori then it is possible to include this in the identification formulation. The problem now reduces to finding a model that minimizes *γ *while ensuring that it accounts for the uncertain data. A model satisfying these conditions is said to be *valid *for that dataset and should have *γ *= 0.

### Model Invalidation

We now turn to the problem of model invalidation given experimental data. Given the data points (*t*_*i*_, ) for *i *= 1,..., *N *where  and a model of the form (1) or (2) with parameters that can either be fixed or *p *∈ , our task is to show that the model can not represent the data. Note that in order to invalidate a model, one data point is enough apart from the initial time *t*_1 _– assume this has been chosen to be at *t*_ℓ _where ℓ ∈ {2,..., *N*}. The aim is to show that this experimental observation could not have arisen from the set of models that are being considered. In cases where it is not clear which point ℓ ∈ {2,..., *N*} to choose, it is suggested that the point with the largest residual (between the nominal model and data) is chosen. As already mentioned, the sets  and  are assumed to be described by polynomial inequalities, eg,  where *g*_*i *_are polynomial functions.

#### Continuous-time case

For invalidating nonlinear continuous-time models with parametric uncertainty given experimental data, we can use a method similar in concept to that of constructing a Lyapunov function to establish equilibrium stability: Lyapunov functions ensure the stability property by making sure that trajectories do not escape their sub-level sets. In [25] the related concept of *barrier certificates *is introduced. These are functions of state, parameter and time, whose existence proves that the candidate model is invalid given a parameter set and experimental data, by ensuring that the model behaviour does not intersect the set of experimental data. In this paper we describe some practical aspects of using Barrier functions and develop a parametrization that is more efficient for practical applications. These barrier certificates can be constructed efficiently using Semidefinite Programming and SOSTOOLS.

Consider a system of the form (1) and assume that . Given this information, if it can be shown that for all possible system parameters *p *∈  the model cannot produce a trajectory *x*(*t*) such that *x*(*t*_1_) ∈ , *x*(*t*_ℓ_) ∈  and *x*(*t*) ∈  for all *t *∈ [*t*_1_, *t*_ℓ_], then the model and parameter set are invalidated by .

**Theorem 1 **[[15], *Theorem 2] Given the candidate model (1) and the sets *, *suppose there exists a real valued function B*(*x*, *p*, *t*) *that is differentiable with respect to x and t such that*



*Then the model is invalidated by *. *We refer to the function B*(*x, p, t*) *as a barrier certificate*.

The above theorem requires the construction of a function that satisfies certain non-negativity conditions, which is not easy. If we relax these constraints to sums of squares, then this computational relaxation can be used to construct barrier certificates. At the same time, this imposes that *B *will be polynomial in the states, parameters and time variables. This may make sense for states and parameters, but a more natural choice for the dependence of *B *on time is of the form *e*^-*λt *^for some *λ*. This is because the Barrier function *B *resembles in shape system trajectories and therefore this choice for time-dependence is more appropriate. The value of *λ *can be chosen, eg, based on the characteristic time constant of the system. Denoting *μ *= *e*^-*λt *^for fixed *λ*, we seek a *B *as a polynomial in (*x*, *p*, *λ*) with real coefficients *c*_1_,..., *c*_*m*_:

(7)

where *b*_*j*_(*x*, *p*, *μ*)'s are monomials in *x*, *p*, *μ*. The SOS program is then a search for the coefficients *c*_*j*_'s such that the conditions stated in Theorem 1 are met. Note that the time re-parametrization will change the derivative condition in the theorem above, as we will see in the sequel, to:



Where  and .

For concreteness, define the sets  and  as

(8)

(9)

(10)

(11)

where the *g*'s are polynomials written in the format described by (3).

The first condition in Theorem 1 states that *B*(*x*_ℓ_, *p*, ) - *B*(*x*_1_, *p*, ) must be positive. As a SOS decomposition provides a polynomial that is *nonnegative *and not positive, a small positive scalar *" *must be subtracted before the constraint is asked to be a SOS.

In order to include the constraints in the formulation, we need to adjoin them to the two conditions given in Theorem 1. This can be done using Lagrange-type multipliers, for the first condition we denote the multipliers with an *M*: *M*_*P*, *i*_(*x*_1_, *x*_ℓ_, *p*) for all *g*_*pi*_(*p*), *M*_1, *i*_(*x*_1_, *x*_ℓ_, *p*) for all *g*_1, *i*_(*x*_1_) and *M*_ℓ, *i*_(*x*_*l*_, *x*_ℓ_, *p*) for all *g*_ℓ*i*_(*x*_*i*_), while the multipliers for the second condition are denoted with by an *N*. All of the multipliers have to be nonnegative (SOS), as they are used to adjoin inequality constraints to the optimization problem. The SOS optimization problem can now be stated as:

(12)

(13)

If a solution to the optimization problem described by (12)–(13) can be found that satisfies all the constrains (8)–(11) then a barrier certificate *B*(*x*_ℓ_, *p*, *μ*_*t*ℓ_) has been constructed which proves that the model is invalid as per Theorem 1. A solution to the optimization problem will provide the coefficients (*c*_*j*_'s) which make expressions (12)–(13) and *M*_*P*, *i*_, *M*_1, *i*_, *M*_ℓ*i*_, *N*_*P*, *i*_, *N*_*X*, *i *_for all *i *and *N*_*t *_sums of squares and invalidates the model and the parameter set by . In the final section results are shown for barrier certificates constructed using the ordinary and new time parametrization method.

#### Discrete-time case

Given the discrete-time model of the form (2) one can develop a relationship between *x*_ℓ _and *x*_1_:



which we write in short



Since *f *was assumed to be polynomial, *F*_ℓ _is also polynomial and the problem of model invalidation can be formulated as the emptiness of the following set:



The sets ,  and  are assumed to be described by polynomial inequalities as described in the previous section, in which case invalidation is equivalent to



where *h*(*x*_1_, *x*_ℓ_, *p*) = *F*_ℓ_(*x*_1_, *p*) - *x*_ℓ_. Testing emptiness of the latter can be done in many ways, the simplest of which is to construct multipliers *σ*_*i*_(*x*_1_, *x*_ℓ_, *p*) that are Sum of Squares and *λ*(*x*_1_, *x*_ℓ_, *p*) polynomial so that

(14)

To see why this is so, suppose there is a point in the set we want to show is empty, ie, a point (*x*_1_, *x*_ℓ_, *p*) that satisfies the inequalities *g*_*i*_(*x*_1_, *x*_ℓ_, *p*) ≥ 0 and *h*(*x*_1_, *x*_ℓ_, *p*) = 0. Then the above condition says that something negative is a SOS, a contradiction. In this case the multipliers form a certificate that the set is empty, ie, that the model is invalidated by the data. These certificates can be constructed via convex optimization and sum of squares programming, as explained in the previous section. The above formulation is a special case of Positivstellensatz, a central theorem in real algebraic geometry – for more details see [26].

## Results

We now present examples that illustrate how the methods described in the previous section can be used. We first consider a simple biochemical reaction network, that of an enzymatic reaction with product degradation. The second example demonstrates the methodology previously described for invalidating a discrete time model.

### Continuous-time: Biochemical Reaction Network

A simple biochemical reaction network with a product degradation term is given by:

(15)

The enzyme, *E*, binds reversibly with the substrate, *S*, to form a complex *ES *which then forms a product *P *and releases the enzyme *E*. The parameters on the arrows denote the rate constants and are used to quantitatively describe the speed of a reaction in a given direction. Denoting the concentration of the reactants by lowercase letters where [·] denotes concentration and *e *= [*E*], *p *= [*P*], *s *= [*S*] and *c *= [*ES*], the law of mass action results in the system of equations:

(16)

(17)

(18)

where *e*(*t*) can be calculated from the conservation relation *e*(*t*) = *e*(*t*_0_) -*c*(*t*). The above system is initialized from *s*(*t*_0_) = *s*_0_, *c*(*t*_0_) = 0 and *p*(*t*_0_) = 0. The reaction rates and *e*(*t*_0_) are assigned values as described later in this section.

Suppose a model for the above system has been proposed that takes the form

(19)

(20)

This model structure is very commonly used in practice to describe the chemical reaction network described by (15). In fact, the appropriate parameter choice should be *V *= *k*_2_*e*_0_,  and *λ *= *k*_3 _if a singular perturbation of the *c *dynamics in (16) – (18) were allowed.

The original model (16–18) is simulated to generate *experimental *data, for the purposes of this example. The parameters are fixed to *k*_1 _= 4.5(nM s)^1^, *k*_-1 _= 2.5s^-1^, *k*_2 _= 56s^-1^, *k*_3 _= 2s^-1 ^and the initial condition to *e*_0 _= 3.5 nM. We first follow the approach described previously to find a lower bound on the error between the model predictions and experimental data, and then use the continuous-time approach that we described to invalidate this model.

The error between the model predictions and the experimental data is given by:

(21)

Here, the following (Euler) discretized version of the model described by (19)–(20) is used



where Δ*t *denotes the discretization time step, which is equal to the sampling rate of the experimental data. It should be noted that the Euler discretization has been chosen for simplicity. There are numerous alternative methods of discretizing the system (eg, central difference approximation).

A lower bound on the minimum value that the expression (21) can achieve can be found using the sum of squares decomposition, as described in the Methods section. Reformulating (21) to remove the denominator we require that

(22)

where *N *is the number of samples,  and



The optimization objective is now to maximize *γ*, which represents the lowest bound achievable for the difference between model predictions and observed data. Once such a value for *γ *has been obtained and is exact, we can substitute this back into the original equation and solve for p* as described in the methods section.

**Figure 1:**
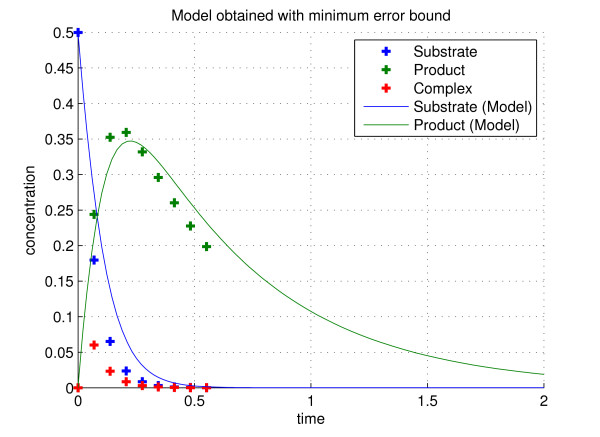
**Simulated experimental data (dashed points) and the minimum error bounded model (smooth curves)**. In this figure the first 9 data points from the experimental data obtained from (16–18) are represented by the colored dashes. The complex, substrate and product concentrations are denoted by the red, blue and green dashes respectively. Shown in comparison to this are the data obtained from the minimum error bounded model (19–20), the green curve represents the product concentration and the blue curve the substrate concentration.

We used the method described above to find that the lower bound on the error between data and the predictions of the model given by (19)–(20) is *γ *= 0.00328. This was done using nine data points and the optimal parameter values that give this error are *K *= 1.313, *V *= 14.856 and *λ *= 1.736. The behavior of the system for this set of parameters is shown in Figure 1, plotted against the experimental data. There, it is evident that the model has captured adequately the dynamics of the system – the fast transient responses have been reflected in the model data.

**Figure 2:**
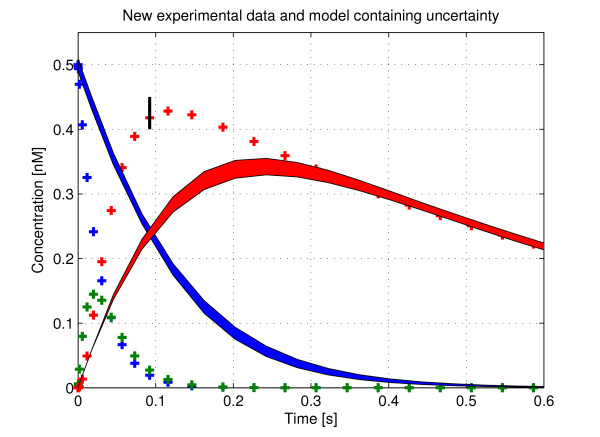
**Experimental data (dashes) and model data with unknown initial conditions in the substrate (blue) and parametric uncertainty in *V***. Simulated experimental data using the new parameter set from the model described by (16–18) are plotted with the complex, substrate and product shown in red, blue and green respectively. All possible trajectories of the product and substrate (given the uncertain sets described) from the model (19–20) are shown by the solid red and blue patches respectively. We aim to invalidate the model with the data at *t *= 0.0923 where the experimental data contains uncertainty as indicated by the black error bar.



We now focus on the validity of the model given the parameters obtained. For this, a second data set is simulated for the purpose of invalidation. The new *experimental *data is obtained by simulating model (16–18) in the same manner as before with *k*_1 _= 8.5(nM s)^-1^, *k*_-1 _= 2.5s^-1 ^and *e*_0 _= 4.5 nM with the remaining parameters unchanged. We also consider an uncertain model of the system (to account for possible experimental error in the original data set) and we assume that we do not have an exact value for *V *(in [nM]/s) but rather we are confident that *V *lies in the interval *V *∈ [13,15]. Uncertain initial conditions in the substrate concentration [*S*] are introduced such that *s*(*t*_0_) = *s*_0 _∈ [0.49, 0.51] and finally, the time point chosen to invalidate the model at is *t*_ℓ _= 0.0923 where the product data due to experimental error lies in the range *p*(*t*_ℓ_) = *p*_ℓ _∈ [0.4, 0.45]. We choose to attempt the invalidation at this point because the error between the nominal model prediction and the data point is very large as can be seen in Figure 2. For high order nonlinear models invalidation through visual inspection of all possible trajectories is not possible. Once it has been shown that the model and data do not agree at a single time point the model is said to be invalid. Describing the uncertainty in the form of constraints using the notation of (8)–(11) we have:

In this instance we look for a barrier certificate of the form *B*(*x*, *V*, *t*) ie, a polynomial function of state, parameter and time. This is a more demanding problem than the nominal system invalidation problem as we are dealing with uncertain data, as well as model uncertainty in the form of unknown initial conditions and parameters. We aim to invalidate the model based on experimental observations from the product of the reaction alone.

The algorithm is able to construct a barrier certificate that is bounded by monomials of maximum degree 4 in state and first degree in time and parameter (the barrier certificate obtained is given in the online Additional File 1). The existence of a barrier certificate tells us that given the observed experimental data at *t *= 0 and *t *= *t*_ℓ _the proposed model is not able to replicate the observed data given these initial conditions and parameter sets, hence it is invalidated. For comparison we now seek to find a barrier certificate of the form *B*(*x*, *p*, *μ*) where *μ *= *e*^-*λt*^. Setting *λ *= 0.8 the algorithm is able to find a lower order certificate, in this case the barrier certificate is bounded by monomials of the state vector of degree three. We have shown for this example that the time re-parametrization can provide a more efficient means for constructing barrier certificates.

#### Discrete-time: Population Growth

We now give an example of discrete model invalidation using the formulation shown previously. We use a delayed logistic equation to model single species growth with after effect to generate *experimental *data:

(23)

where *r *∈ [1.5, 2]. The model that is being considered ignores the delay and takes the following form:

(24)

The only free parameter in this model is *r *and we shall constrain it such that *r *∈ [1.5, 2].

Our aim is to show that given a set of experimental data we can provide an invalidation certificate for our model.

**Figure 3:**
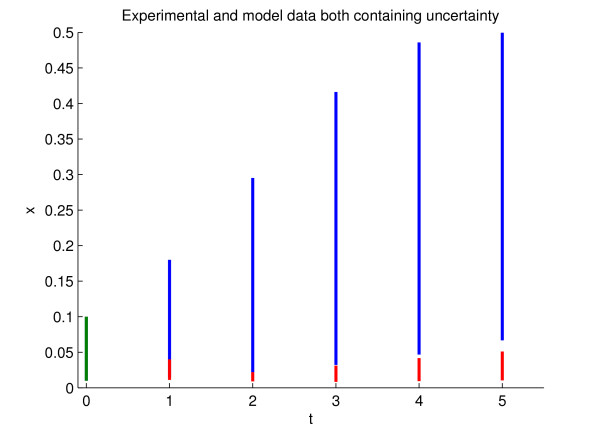
**Discrete time logistic growth model**. Data ranges for the simulated experimental data (red) generated from (23) and model data (blue) from (24) given parametric and initial condition uncertainty. The first green bar indicates that both data sets start from the same unknown initial condition set.

A plot of the behaviour of the two models given the parameter and initial condition ranges is shown in Figure (3). Model invalidation amounts to ensuring that at some point the set of possible model behaviours and the experimental data set do not intersect given the uncertainty. Examining Figure (3) it is clear that at *k *= 3 and *k *= 4 the intersection of the two sets is empty, however this is also true for *k *= 2. Normally when a model has multiple parameters or is nonlinear of high dimension it may not be possible analytically or through simulation to see where this occurs. This reason alone highlights the importance of our method. For this example we have *r *∈ [1.5, 2] and *x*_0 _∈ [0.01, 0.1] (assuming that the delayed model has constant initial conditions) and at *k *=2,  where *x *is the experimental data at that point in time and *x*_2_ = 0.00225,  = 0.016384. We therefore define the following sets:

(25)

In this case, the invalidation problem becomes

(26)

where



The test described by (14) becomes

(27)

where *s*_*i *_are sums of squares and *t *is a polynomial. The degree of the unknown polynomial *t *and the SOS variables *s*_*i *_has to be chosen carefully in order to make sure that the computational effort is acceptable. The above SOS program was implemented using SOSTOOLS and the model was successfully invalidated based on the experimental data at *k *= 2. The degree of the multipliers was deg{*s*_1_} = 2, deg{*s*_2_} = 2, deg{*s*_3_} = 2 and deg{*t*_1_} = 1:



As we can see from the above, the multipliers certifying the emptiness of set (26) are large, indicating the sensitivity of the certificate to changes in the model structure and parameters. This should be expected, as the fact that the model can be invalidated using data at *k *= 2 is not immediately obvious by looking at Figure (3). The computational effort needed to invalidate the model increases as data for larger *k *are used in the invalidation, since the polynomial *F*_*k *_in (26) becomes more complicated. However, the coefficients of the multipliers become smaller, indicating that the certificate robustly verifies that the model is invalid.

## Discussion

In this paper we have presented a method for calculating a bound on the error between predictions from an ODE model of a particular structure and experimental data. We introduced an error metric *γ *which is used to quantify a lower bound on this difference. Note that *γ *is actually an error metric in the same way the sum of squares error (SSE) is commonly used for fitting models to data: for that reason it should only be used to compare the goodness of fit in different scenarios, eg, comparing competing model structures or the same model structure for different data sets. Ideally *γ *should be as low as possible.

An alternative error metric that may be worth investigating in the future is the maximum difference between the model prediction and all data points. With this method, normalization of *γ *is not required. This error metric gives us additional information on where the model predictions are weakest, which could be used to automatically select the instance at which to begin the invalidation process.

As mentioned already, our methodology for identification and invalidation uses the fact that we can quantify the level of uncertainty in the experimental data and using this information, develop a parameterized, uncertain model that can be used to describe the data set. In fact, the researcher that conducted the experiments should be able to provide upper and lower bounds on measurements, which can then be used for building the uncertain sets . At the same time, the set  should ensure that the uncertain model can describe every point in the data set that was used to fit it. This set could also be used to encompass all the uncertainty present in the system, including environmental fluctuations, experimental error and measurements that prove difficult to obtain accurate measures for. See [4] for more details on how the set  can be constructed.

Traditionally, models are fitted on one data set and a second, unseen data set can be used to either validate it (and refine its parameters), or invalidate it. Assume that we have fitted a model of the form  = *f*(*x*, *p*), from some experimental data set *x *∈  and obtained the parameter set *p *∈  using the error bounding method described in the Methods section. Given a second data set, we would like to know how well our model describes these new data. This can be formulated as an optimization problem that seeks to find a parameter value , so that the new model  = *f*(*x*, *q*) can represent the new data set. The size of ||*δp*|| = ||*q *- *p*||, reflects the magnitude of the change required in the parameter values in order for the model to replicate the new data. If ||*δp*|| is large then this indicates that the original model does not accurately describe the system under study. If ||*δp*|| is not large and , then we can refine the model parameters appropriately by grouping the two data sets. Otherwise, we can formally invalidate the old model and seek a different structure in the dynamics of the system under consideration, using eg, the approach in [27]. To this end, ||*δp*|| provides a measure of the sensitivity of the model parameter set to new experimental data. This cyclic approach will then lead to a better understanding of the system under study as it can also be used to identify what in the model structure fails to replicate the data.

## Conclusion

We have demonstrated how questions related to system identification and model invalidation for biological systems can be answered using optimization, real algebraic geometry and dynamical systems concepts. We have emphasized that formal model invalidation in conjunction with system identification can be used to develop reliable models of biological systems.

Finding a lower bound on the error during an identification approach is important information that can not only be used to provide feedback as to the suitability of the model structure chosen, but also can help in identifying the suitable parameters, as demonstrated in the results section. More complicated model structures can also be used, even if these are not polynomial, through appropriate recasting [28].

One of the biggest problems encountered when trying to construct a barrier certificate (for continuous-time systems) and appropriate multipliers (for discrete-time systems) is determining the minimum order of the polynomials that would allow invalidation. For complicated system descriptions, constructing such polynomials can be computationally demanding, but the problem still remains polynomial-time verifiable. This is in contrast to simulation-based approaches that require an exponential number of runs and anyway cannot always answer the invalidation question conclusively. The examples given in this paper aim to introduce the algorithms that can be used for model invalidation and were hence kept simple for clarity-the algorithmic formulations are suitable for more complicated examples.

A future direction will be to investigate what information barrier certificates and multipliers (for invalidation of continuous and discrete time systems respectively) can provide about the invalidated model – ie, what system structure could be changed in order for such a certificate not to exist.

## Authors' contributions

JA developed the theory, implemented the examples and wrote the paper. AP conceived the general idea of using SOS decompositions for discrete model invalidation and error estimation, and suggested the reparametrization in the continous-time case.

## Supplementary Material

Additional File 1 **Barrier certificate**. This PDF file details the Barrier Certificate obtained by solving the optimization problem (12)–(13) that invalidates the biochemical reaction model. Click here for file

## References

[B1] Lander AD (2004). A calculus of purpose. PLoS Biol.

[B2] Murray JD (2002). Mathematical biology, I: An Introduction.

[B3] Ljung L (1999). System Identification: Theory for the User.

[B4] Frenklach M, Packard A, Seiler P, Feeley R (2004). Collaborative data processing in developing predictive models of complex reaction systems. Int J Chem Kinet.

[B5] Feng X, Rabitz H (2004). Optimal Identification of Biochemical Reaction Networks. Biophys J.

[B6] Smith RS, Doyle JC (1992). Model Validation: A connection between robust control and identification. IEEE Trans Automat Contr.

[B7] Beck M, Ravetz J, Mulkey L, Barnwell T (1997). On the problem of model validation for predictive exposure assessments. Stochastic Hydrology and Hydraulics.

[B8] Kremling A, Fischer S, Gadkar K, Doyle FJ, Sauter T, Bullinger E, Allgöwer F, Gilles ED (2004). A Benchmark for Methods in Reverse Engineering and Model Discrimination: Problem Formulation and Solutions. Genome Res.

[B9] Chen BH, Asprey SP (2003). On the Design of Optimally Informative Dynamic Experiments for Model Discrimination in Multiresponse Nonlinear Situations. Ind Eng Chem Res.

[B10] Gadkar KG, Gunawan R, Doyle FJ (2005). Iterative approach to model identification of biological networks. BMC Bioinformatics.

[B11] Newlin MP, Smith RS (1998). A generalization of the structured singular value and its application to model validation. IEEE Trans Automat Contr.

[B12] Smith RS, Dullerud G (1996). Continuous-time Control Model Validation Using Finite Experimental Data. IEEE Trans Automat Contr.

[B13] Polisetty PK, Voit EO, Gatzke EP (2006). Identification of metabolic system parameters using global optimization methods. Biol Med Model.

[B14] Parillo P (2003). Semidefinite programming relaxations for semialgebraic problems. Mathematical Programming Ser B.

[B15] Prajna S (2006). Barrier certificates for nonlinear model validation. Automatica.

[B16] El-Samad H, Prajna S, Papachristodoulou A, Doyle J, Khammash M (2006). Advanced methods and algorithms for biological network analysis. Proceedings of the IEEE.

[B17] Yi TM, Fazel M, Liu X, Otitoju T, Goncalves J, Papachristodoulou A, Prajna S, Doyle JC (2005). Application of Robust Model Validation using SOSTOOLS to the study of G-Protein Signalling in Yeast. Proceedings of FOSBE.

[B18] Mitchell IM, Bayen AM, Tomlin CJ (2005). A time-dependent Hamilton-Jacobi formulation of reachable sets for continuous dynamic games. IEEE Trans Automat Contr.

[B19] Murty KG, Kabadi SN (1987). Some NP-complete problems in quadratic and nonlinear programming. Mathematical Programming.

[B20] Prajna S, Papachristodoulou A, Seiler P, Parrilo PA (2004). SOSTOOLS: Sum of squares optimization toolbox for MATLAB.

[B21] Sturm JF (1992). Using SeDuMi 1.02, a MATLAB toolbox for optimization over symmetric cones. Optimization methods and software.

[B22] Kuepfer L, Sauer U, Parrilo PA (2007). Efficient classification of complete parameter regions based on semidefinite programming. BMC Bioinformatics.

[B23] Dullerud GE, Paganini FG (2000). A course in robust control theory: A convex approach.

[B24] Zhou K, Doyle J, Glover K (1996). Robust and optimal control.

[B25] Prajna S (2005). Optimization-based methods for nonlinear and hybrid systems verification. PhD thesis.

[B26] Parrilo PA (2000). Structured Semidefinite Programs and Semialgebraic Geometry Methods in Robustness and Optimization. PhD thesis.

[B27] August E, Papachristodoulou A (2009). Efficient, sparse biological network determination. BMC Syst Biol.

[B28] Papachristodoulou A, Prajna S (2005). Analysis of Non-polynomial Systems Using the Sum of Squares Decomposition. Positive Polynomials in Control.

