# Supplementary material for: On validation and invalidation of biological models
Source: BMC Bioinformatics. 2009 May 7;10:132. doi: 10.1186/1471-2105-10-132 (PMC2704209; doi:10.1186/1471-2105-10-132)
Supplement: Additional File 1 — Barrier certificate. This PDF file details the Barrier Certificate obtained by solving the optimization problem (12)–(13) that invalidates the biochemical reaction model. [file 1471-2105-10-132-S1.pdf]

On Validation and Invalidation of Biological Models

James Anderson[*] Antonis Papachristodoulou[†]

Additional File 1: Barrier Certificate

The Barrier certificate obtained by solving the optimization problem in equations (12)-(13) of the main text:

$$\begin{aligned}
B(x,V,t) =& 0.0107x_1x_2t + 0.372x_2V + 0.798 \times 10^{-3}x_1Vt + 0.231 \times 10^{-2}tx_1^4 + 0.218 \times 10^{-2}tx_2^3x_1 + \\
& 0.482 \times 10^{-3}x_2Vt + 0.322 \times 10^{-4}tx_2^4x_1 + 0.521 \times 10^{-2}tx_1^3 + 0.578 \times 10^{-4}tx_1^3x_2^2 + \\
& 0.132x_1^5 + 0.0918x_2^5 + 0.654 \times 10^{-3}x_1tVx_2^2 + 0.330x_2x_1^2 - 0.643 \times 10^{-4}x_2^3tV - \\
& 0.241 \times 10^{-2}x_2^2x_1t + 0.564 \times 10^{-4}x_1tVx_2^3 + 0.0170Vx_1^2 + 0.0151x_2^2x_1^2 - 0.249 \times 10^{-2}tx_2Vx_1 + \\
& 0.408Vx_2^2 + 0.635x_1x_2^2 - 0.555Vx_1^3 - 0.203x_2x_1^3 - 0.663 \times 10^{-3}tVx_2^2 - 0.551 \times 10^{-3}tx_2^3 - \\
& 0.536 \times 10^{-3}tx_1^4x_2 + 0.443Vx_1^3x_2 + 0.197 \times 10^{-3}tx_2x_1^2V + 0.0628Vx_2^3 + 0.459 \times 10^{-3}tx_2^4 + \\
& 0.0618x_1x_2^3 - 0.114 \times 10^{-3}tx_1^5 + 0.177 \times 10^{-3}tx_2^3x_1^2 + 0.0187x_1Vx_2^2 - 0.969 \times 10^{-2}x_1Vx_2^3 + \\
& 0.769Vx_2^2x_1^2 + 1.30x_1^2Vx2 - 0.584x_1Vx_2 + 0.654x_2x_1^4 + 0.459x_1x_2^4 + 0.076x_1 + 0.238x_2 - \\
& 0.246 \times 10^{-2}Vx2^4 + 0.274 \times 10^{-2}x_1^2t + 0.859x_2^2x_1^3 + 0.800x_2^3x_1^2 - 0.005x_2t + \\
& 0.996 \times 10^{-4}tVx_1^3 - 0.522x_1x_2 + 0.402 \times 10^{-2}tx_2x_1^3 - 0.157 \times 10^{-4}tx_2^4V + 0.877 \times 10^{-2}tx_2x_1^2 - \\
& 0.728 \times 10^{-3}tVx_1^2 + 0.387 \times 10^{-3}tx_1^2Vx_2^2 + 0.414x_1V + 0.317 \times 10^{-2}tx_2^2x_1^2 - \\
& 0.185 \times 10^{-2}x_2^2t - 1.43x_2^2 + 0.949Vx_1^4 - 0.258x_1^2 - 0.0117x_1t - 0.326 \times 10^{-4}tx_1^4V - \\
& 0.447 \times 10^{-5}x_1^5V + 0.166x_1^3 - 0.0362x_1^4 + 0.382x_2^3 + 0.0928x_2^4 + 0.251 \times 10^{-3}tVx_2x_1^3 + \\
& 0.646 \times 10^{-5}tx_2^5
\end{aligned}$$

[*]james.anderson@dtc.ox.ac.uk
[†]antonis@eng.ox.ac.uk
